# Systematic Review of Behaviour Change Techniques within Interventions to Reduce Environmental Tobacco Smoke Exposure for Children

**DOI:** 10.3390/ijerph17217731

**Published:** 2020-10-22

**Authors:** Tracey J. Brown, Sarah Gentry, Linda Bauld, Elaine M. Boyle, Paul Clarke, Wendy Hardeman, Richard Holland, Felix Naughton, Sophie Orton, Michael Ussher, Caitlin Notley

**Affiliations:** 1 Norwich Medical School, University of East Anglia, Norwich NR4 7TJ, UK; sarah.gentry@doctors.org.uk (S.G.); paul.clarke@nnuh.nhs.uk (P.C.); c.notley@uea.ac.uk (C.N.); 2 Usher Institute and SPECTRUM Consortium, College of Medicine and Veterinary Medicine, University of Edinburgh, Edinburgh EH8 9AG, UK; linda.bauld@ed.ac.uk; 3 Department of Health Sciences, University of Leicester, Leicester LE1 7RH, UK; eb124@leicester.ac.uk; 4 Neonatal Intensive Care Unit, Norfolk and Norwich University Hospitals NHS Foundation Trust, Norwich NR4 7UY, UK; 5 School of Health Sciences, University of East Anglia, Norwich NR4 7TJ, UK; w.hardeman@uea.ac.uk (W.H.); f.naughton@uea.ac.uk (F.N.); 6 Leicester Medical School, University of Leicester, Leicester LE1 7HA, UK; rch23@leicester.ac.uk; 7 Division of Primary Care, University of Nottingham, Nottingham NG7 2RD, UK; sophie.orton@nottingham.ac.uk; 8 Population Health Research Institute, St George’s, University of London, London SW17 0RE, UK; mussher@sgul.ac.uk; 9 Institute for Social Marketing and Health, University of Stirling, Stirling FK9 4LA, UK

**Keywords:** systematic review, behaviour change techniques, smoking, harm reduction, second-hand smoke, tobacco smoke pollution, postnatal, children

## Abstract

Children are particularly vulnerable to environmental tobacco smoke (ETS). There is no routine support to reduce ETS in the home. We systematically reviewed trials to reduce ETS in children in order to identify intervention characteristics and behaviour change techniques (BCTs) to inform future interventions. We searched Medline, EMBASE, CINAHL, PsycINFO, ERIC, Cochrane Central Register of Controlled Trials, and Cochrane Tobacco Addiction Group Specialised Register from January 2017 to June 2020 to update an existing systematic review. We included controlled trials to reduce parent/caregiver smoking or ETS in children <12 years that demonstrated a statistically significant benefit, in comparison to less intensive interventions or usual care. We extracted trial characteristics; and BCTs using Behaviour Change Technique Taxonomy v1. We defined “promising” BCTs as those present in at least 25% of effective interventions. Data synthesis was narrative. We included 16 trials, of which eight were at low risk of bias. All trials used counselling in combination with self-help or other supporting materials. We identified 13 “promising” BCTs centred on education, setting goals and planning, or support to reach goals. Interventions to reduce ETS in children should incorporate effective BCTs and consider counselling and self-help as mechanisms of delivery.

## 1. Introduction

Smoking has a severe detrimental impact on parental and child health [1]. Exposure to environmental tobacco smoke (ETS) from parents or caregivers increases rates of sudden infant death syndrome, respiratory conditions, and other infections [2]. Children are more susceptible to second-hand smoke than adults are [3,4], particularly vulnerable children, such as premature infants [5]. Exposure to smoke in early life results in increased morbidity throughout childhood and into adulthood [2,6,7]. Children exposed to tobacco smoke in utero or in early life are more likely to be admitted to paediatric care, or to a neonatal intensive care unit (NICU) [8,9], resulting in significant economic burden [10,11,12,13]. In the U.K., the annual cost of smoking in pregnancy is estimated to be £64 million for treating maternal health problems and a further £23.5 million for treating infants [14]. Pregnant women and parents are motivated to quit smoking for the health of their children [15,16], but smoking relapse rates are high [17], particularly post-birth [18,19]. Living with a smoking partner or other smoking household member and stress, which may arise from increased parenting demands or lack of sleep, increase the likelihood of relapsing to smoking postpartum [20]. Smoking prevalence is also higher in lower socioeconomic groups [1]. For parents able to remain abstinent and for never smokers, maintaining a smoke-free environment is still challenging when there are other family or household members who smoke [15].

The birth of a child offers a “teachable moment” to support smoke-free environments [21,22,23]. National guidance recommends support for smoke-free strategies in secondary care settings during pregnancy and after childbirth [24,25,26]. However, interventions to maintain smoke-free environments are not routinely offered in paediatric settings or in the home environment [26,27,28]. Support is particularly limited for very vulnerable children, such as those admitted to a NICU, where support to maintain a smoke-free environment is especially crucial [22,29]. Evidence of effective interventions to reduce environmental tobacco smoke (ETS) in young children is limited. A review of smoking cessation in pregnancy and into the postpartum period [19] found some evidence for success of counselling, health education, and incentives, for 0 to 17 months postpartum, but no effect beyond this. A systematic review of interventions to reduce tobacco smoke pollution in homes found that, overall, interventions trialled did improve tobacco smoke air pollution but did not link effectiveness to “type” of intervention [30]. A Cochrane review [27], determining the effectiveness of reducing exposure of children aged 0 to 12 years to ETS, found a minority of interventions reduced exposure, and the features that differentiated effective from ineffective interventions remain unclear [27]. Behaviour change interventions are complex by nature, comprising multiple components such as mechanisms of delivery in addition to behaviour change techniques (BCTs) [31]. By identifying BCTs within effective interventions, it may be possible to specify what components might be combined to develop more successful interventions [32]. No previous reviews have identified BCTs to reduce ETS exposure in young children or have drawn firm conclusions of effective mechanisms of delivery. Behbod et al. [27] conducted literature searches up to February 2017, and updating this review might identify new and effective interventions. We aimed to systematically review controlled trials aiming to reduce the ETS exposure of children aged under 12 years, to identify promising mechanisms of intervention delivery and BCTs to inform future interventions. Our review was registered on the Open Science Framework on 23 May 2019 and was updated on 22 January 2020 (https://osf.io/zhmtu/).

## 2. Materials and Methods

### 2.1. Approach

This systematic review is guided by the Preferred Reporting Items for Systematic Reviews and Meta-Analyses (PRISMA) guidelines [33]. First, we updated an existing systematic review of controlled trials to reduce children’s exposure to ETS [27]. We then identified interventions with evidence of a statistically significant positive effect from identified trials. Finally, we identified BCTs [32] described within these effective interventions.

### 2.2. Search Strategy

We searched Medline (OvidSP), EMBASE (OvidSP), CINAHL (EbscoHOST), PsycINFO (OvidSP), ERIC (ProQuest), Cochrane Central Register of Controlled Trials, and the Cochrane Tobacco Addiction Group Specialised Register from 1 January 2017 to 11 June 2020. We replicated the search strategy used by Behbod et al. to update their systematic review [27]. Keywords included the following: parent, caregiver, family, house, home, newborn, infant, child, tobacco, smoking, smoking cessation, environmental pollution, and tobacco smoke pollution. The full search strategy is published in Behbod et al. [27]. Effective trials published prior to 2017 were identified by handsearching Behbod et al. [27]. Reference lists of included trials were also searched for any relevant articles. We attempted to contact authors of all included trials to collect all published or unpublished details of the intervention methodology and any further trial evaluation data (e.g., study acceptability or feasibility).

### 2.3. Trial Selection

We included controlled trials (randomised and non-randomised as in Behbod [27]) to reduce the ETS exposure of families with young children. Participants were parents or caregivers of children aged under 12 years of age. We included trials where the primary aim was to either reduce children’s exposure to ETS, or reduction or cessation of parent or caregiver smoking, versus another intervention or usual care. We included trials with a follow-up period of 6 months or more. Since our focus was on interventions for parents or caregivers that would be suitable to use in any child under 12 years, we excluded trials which included any child ≥12 years, or trials in which children undertook any intervention activities themselves (e.g., parent/child dyads), or trials that included school-based (or other educational establishment) intervention activities. Trials not published in English were also excluded due to the detailed nature of identifying BCTs [32]. We aimed to identify promising BCTs, thus we included only trials that were “effective” at long-term follow-up (6 months or more from baseline), defined as “a reported statistically significant *p* value of <0.05, with ETS exposure or smoking status of household members as the primary outcome (whether or not biochemically validated)”.

Two authors (two from T.J.B., S.G., or C.N.) independently screened citations on the basis of title and abstract using Covidence software and also using tables of study characteristics published in Behbod et al. when hand-searching [27]. Any disagreements were resolved by consensus. Where it was unclear whether a study met our inclusion criteria, the full text was collected and assessed in duplicate. Each full-text article was assessed for inclusion using an inclusion log within Covidence, and reasons for study exclusion were also recorded.

### 2.4. Data Extraction

Trial characteristics for both the intervention and control groups were extracted into a tailor-made Excel sheet to include the following: trial design, participants, sample size, country, details of the intervention and control procedures, behavioural theory, outcome measures, smoking outcomes, and process indicators. Our smoking outcomes were ETS exposure (as defined by authors), and smoking status of family or household members. Additional outcome measures were acceptability, feasibility, child health outcomes (e.g., respiratory illness, use of health services), and behaviour change (e.g., implementation of a household smoking ban).

We used Behaviour Change Technique Taxonomy v1 (BCTTv1) [32] to extract BCTs from intervention and control descriptions of all included articles (the main paper and associated articles as relevant for each trial). We extracted BCTs that targeted smoking cessation, smoking relapse, or behaviours relating to a reduction of ETS. BCT codes were assigned to relevant sections of articles and were extracted if definitely (coded ++) or probably (coded +) present following BCTTv1 principles (www.bct-taxonomy.com). These principles define a coding of ++ as a “BCT present beyond all reasonable doubt”, and a coding of + as a “BCT present in all probability”. We calculated the frequency of BCTs from intervention groups across all effective trials to identify “promising” BCTs that might improve intervention success. In the absence of a gold standard approach [34], we sought BCTs based on prevalence within intervention groups [35]. We defined “promising” BCTs as those present in at least 25% of effective interventions [36].

Data were extracted independently by two BCTTv1-trained researchers. Researchers met to agree on findings, with any disagreements resolved through discussion or the involvement of a third researcher. We did not undertake any statistical analysis due to the wide range of interventions to reduce environmental tobacco smoke and the diversity in populations, settings and outcomes. Data synthesis was narrative.

### 2.5. Quality Assessment

Two researchers (two from T.J.B., S.G., or C.N.) independently assessed the risk of bias for all included studies. Risk of bias was categorised as high, low, or unclear for the following domains: “random sequence generation”, “allocation concealment”, “incomplete outcome data”, “blinding of participants and personnel”, “blinding of outcome assessment”, and for any other bias (e.g., funding) in accordance with the Cochrane Handbook for Systematic Reviews of Interventions [37]. In addition to assessing each of these domains separately, a judgement of overall risk of bias for each trial was reached by consensus among three reviewers (T.J.B., S.G., and C.N.). Since full blinding of the intervention in these trials is not possible due to the nature of their design, we excluded “blinding of participants and personnel” from our overall risk of bias assessment. For the remaining domains, where at least three out of five domains were at low, unclear or high risk of bias, our overall judgement for risk of bias was low, unclear, or high, respectively. Where at least one domain was at high risk of bias, our overall judgement for risk of bias was automatically downgraded to at least a status of unclear. Any disagreements were resolved through discussion.

## 3. Results

### 3.1. Numbers of Trials

**Figure 1:**
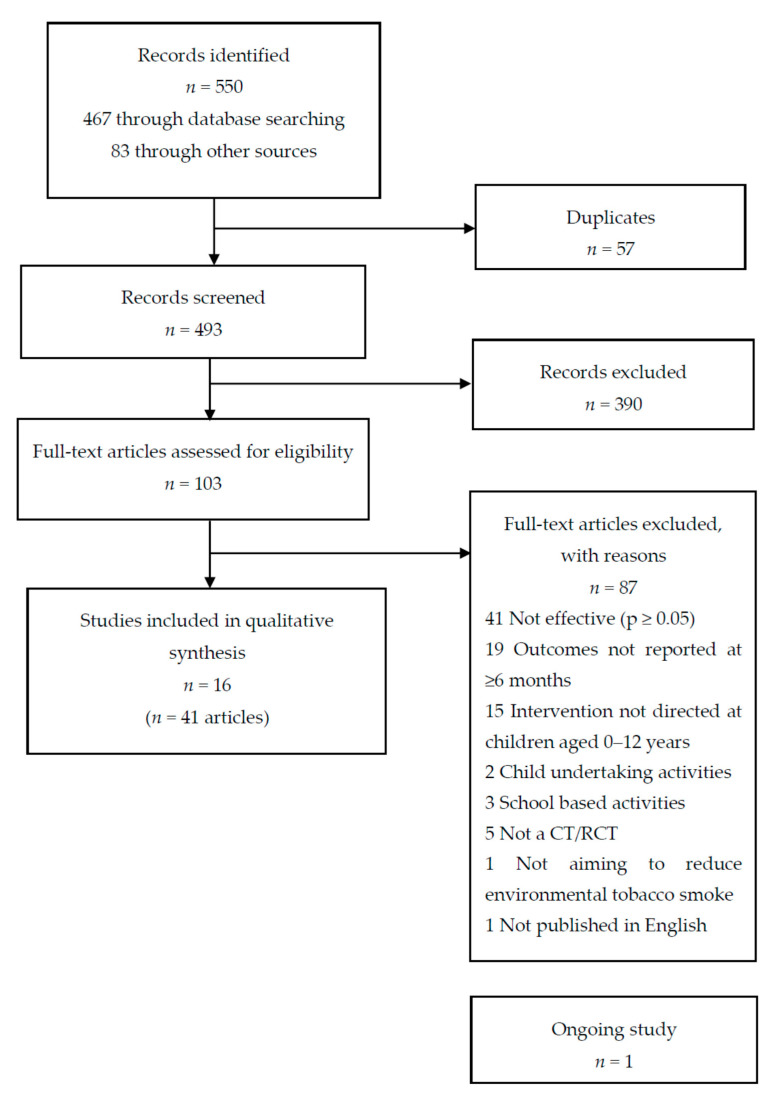
Flow diagram.

The inclusion of controlled trials is shown in Figure 1. Electronic and hand searching identified 550 records, with 493 references remaining after the removal of duplicates. Based on title and abstract screening, 103 relevant articles were retrieved for full-text assessment, with the final inclusion of 16 primary controlled trials [5,38,39,40,41,42,43,44,45,46,47,48,49,50,51,52] (associated with 41 articles, Table S1). Twelve of these trials had previously been identified by Behbod and colleagues [27]. We also identified one relevant ongoing trial [53]. Despite writing to all authors of the included studies, only five responded to our request for further information, of which two supplied information we had not already identified (a published protocol [49]; and a report to study funders [41]).

### 3.2. Trial Characteristics

Fifteen trials aimed to promote smoke-free environments alongside encouraging smoking cessation or abstinence. One trial [44] was designed to promote a smoke-free environment without emphasising smoking cessation or abstinence. Full trial characteristics are shown in Table S2 (including population, sample size, details of the intervention and control, outcome measures, and process indicators). Twelve trials [5,38,39,41,42,44,45,46,47,48,49,52] were randomised controlled trials (RCTs), three were cluster RCTs [40,50,51], and one was a non-randomised controlled trial [43]. Most trials were conducted in the USA [5,40,42,43,44,45,47,48,49,51], with the remaining trials in China [38,39,41,52], Germany [46], and Spain [50]. Six trials were conducted exclusively in neonates [5,43,44,46,51,52], two in young infants (0–18 months) [41,50], five in children aged up to 5 years [38,39,42,47,48], and three in children aged up to 12 years [40,45,49]. Nine trials recruited both parents/caregivers [5,38,39,40,41,42,49,50,52], and seven trials [43,44,45,46,47,48,51] recruited mothers/female caregivers only. Ten trials recruited smokers or recent quitters [38,39,40,42,45,46,47,48,49,50,51], two had mixed populations of non-smokers or smokers [5,44], one focussed on postpartum quitters [43], and two recruited families with a smoking father and non-smoking mother [41,52]. One trial [5] recruited specifically via neonatal intensive care units. Other recruitments were via community health settings [38,39,41,42,47,48,52], hospitals post-delivery [43,44,46,51], paediatric care [49,50], primary care [45], or schools [40]. Five trials [42,45,47,48,49] recruited specifically from low-income or minority group areas.

### 3.3. Intervention Characteristics

Trials used various different theoretical approaches and modes of delivery. Interventions were generally a combination of “counselling” (e.g., motivational interviewing, cognitive behavioural therapy, or counselling based on behaviour change theories) and the provision of self-help or educational materials. Five trials used only this combination [38,39,46,49,50]. Other trials used this combination in conjunction with the provision of nicotine replacement therapy [40,41,48], or the provision of objects or reminders, such as stickers and signs to request a smoke-free environment [43,44,45,47,51]. Two trials provided feedback on smoking outcomes to parents/caregivers as part of the intervention (infant salivary cotinine [5]; or air nicotine, caregiver carbon monoxide levels, and respiratory symptoms [42]) in addition to counselling and self-help materials. One trial added supportive text messages to one of the intervention arms [52]. Control groups received less intensive interventions [39,41,42,46,47,49,51], less smoking information [5,38,40,43,45], or usual care (generally brief advice) [44,48,50,52]. Intervention delivery was usually through a combination of in-person and telephone contacts, but six trials provided counselling in-person [44,45,50,51,52] or by telephone only [39]. Counselling was delivered by nurses [5,39,41,43,44,45], student or graduate counsellors [47,48,49], health workers [38,42,52], primary care staff [50], paediatric staff [51], or general trained counsellors [40,46]. Interventions varied from the provision of a single counselling session [45] to up to 14 sessions [48] (mean 5 sessions). Not all trials reported session lengths, but where reported, session length also differed widely between trials from 2 min [51] up to 45 min [5,38,42,44,46]. Intervention duration varied from 1 month to 2 years, with six trials intervening for 6 months or longer [40,44,48,50,51,52]. There was no clear pattern to indicate which intervention intensity or duration would be the most advantageous. Six included trials measured outcomes at 6 months post-enrolment [38,39,42,43,45,50] and ten measured outcomes beyond 6 months [5,40,41,44,46,47,48,49,51,52], with the longest study [40] assessing outcomes for up to 4 years.

### 3.4. Quality Assessment

Eight studies were considered at low risk of bias [5,39,40,41,42,47,49,51], six at unclear risk [38,44,45,48,50,52], and only two were considered at high risk of bias [43,46]. Blinding of participants and personnel was either at high or unclear risk for all studies and, therefore, overall risk of bias would be higher if we had included this within our assessment. Some trials reported acceptability and/or fidelity concerns, and we considered three trials as having more major acceptability and/or fidelity concerns [41,43,46]. Specifically, these trials reported fidelity issues: practical difficulties in delivering the on-site component of the intervention due to “noisy” and “congested” environments in some clinics [41]; inconsistent delivery of intervention elements, such as nurses being significantly less likely to discuss pharmacological options with abstinent women [43]; and a low adherence to the motivational interview protocol with only 38% of sessions showing good adherence [46]. Many trials failed to adequately report evaluation of feasibility (acceptability, fidelity, and/or other process indicators, e.g., verification of parent self-report), suggesting that more trials may have suffered from feasibility issues. The majority of our included trials included a form of biochemical outcome validation. Most used exhaled carbon monoxide or salivary/urinary cotinine concentration [5,38,39,40,41,42,43,44,47,48,49]. Three of these trials also used air nicotine monitoring [42,47,48]. One trial used only infant hair nicotine concentration [50]. Four trials [45,46,51,52] did not include any biochemical validation.

### 3.5. Behaviour Change Techniques

We identified a wide range of BCTs targeting smoking cessation, smoking relapse, or behaviours relating to a reduction of ETS as summarised in Table 1 and detailed (coded as probably +, or definitely ++ present) for each separate trial in Table S2. The majority of BCTs were delivered to intervention rather than to control groups. The number of BCTs identified in control groups for each trial ranged from 1 [38,40,47] to 3 [41], with an average of 0.5 BCTs. A total of 6 of the 93 BCTs were found in control groups. In comparison, the number of BCTs identified in intervention groups for each trial ranged from 3 [51] to 16 [42,46], with an average of 9 BCTs. Study protocols or description of study designs were available for seven trials (six published [42,44,46,49,50,51], one a study report supplied by authors [41]), and the number of BCTs identified in interventions were higher in these trials. A total of 42 of the 93 BCTs from BCTTv1 were found in interventions, and at least one BCT was present from each of the 16 BCT clusters in intervention groups [32]. Most BCTs in intervention groups were found in the “goals and planning” cluster, which focuses on goal setting, problem solving, action planning, and review of goals.

“Promising” BCTs, using our criterion of occurring in at least 25% of intervention groups (excluding those delivered to both intervention and control groups), were the following: social support unspecified (81%), problem solving (69%), information about health consequences (63%), credible source (56%), goal setting behaviour (50%), action planning (50%), social reward (44%), instruction on how to perform a behaviour (44%), review behaviour goals (38%), adding objects to the environment (31%), behaviour substitution (25%), verbal persuasion about capability (25%), and information about social and environmental consequences (25%). Of these BCTs common to intervention groups, all included more ++ (definitely present) than + (probably present) codes, with the exception of “credible source” and “review behaviour goals”. We are therefore less certain of classifying these two BCTs as “promising”. However, neither of these BCTs were delivered to control groups. Of the “promising” BCTs, only “information about social and environmental consequences”, “instruction on how to perform a behaviour”, and “behaviour substitution” occurred in control groups, but occurrence was at a lower frequency (19%, 6%, and 6%, respectively). The most common BCT delivered to control groups was “information about social and environmental consequences”. We found no distinct pattern in BCTs based on trial variables, such as whether assessment was biochemically validated or not. We also found no clear pattern as to which BCTs would be the best to deliver to different populations.

## 4. Discussion

We included 16 controlled trials that were effective in reducing children’s exposure to ETS. Our review has updated and advanced evidence from Behbod et al. (2018), a Cochrane review of smoking control programmes for reducing exposure to ETS in children aged 0–12 years [27]. These authors did not find a clear link between intervention features and study effectiveness. Similarly, earlier reviews of interventions to promote smoke-free home environments for children aged 0–5 years [54] and a review of routine healthcare interventions to reduce tobacco smoke exposure in children aged 0–12 years [55] concluded that further research was required to identify effective elements of interventions. Rosen et al. [30] found some evidence of benefit for interventions to protect children (0–12 years) from tobacco smoke exposure but did not specify which intervention type was most effective. Our review found that effective interventions all used some form of “counselling” supplemented with self-help or other materials, compared to less intensive “counselling” and fewer support materials in control groups. We did not set out to compare effective with non-effective trials; we aimed to investigate characteristics of intervention and control groups within effective trials, to identify promising mechanisms of intervention delivery. A review of prevention of postpartum smoking relapse, also found that effective trials provided self-help mainly in conjunction with counselling [36]. A systematic review for smoking cessation in pregnancy and into the postpartum period similarly found some evidence for a beneficial impact of counselling and, to a lesser extent, health education [19]. In contrast to our present review, these authors also found a beneficial effect of using incentives. We suggest that interventions using counselling and self-help approaches, potentially in conjunction with other elements, are most likely to be effective. Interventions that we included in our present review were most commonly delivered by health professional counsellors, in-person or by telephone.

No previous reviews have aimed to identify effective BCTs to reduce ETS in young children. We identified 13 “promising” BCTs, which focused on social support from health professionals, goals and planning, information giving from a credible source, and developing strategies to aid smoking cessation, prevent relapse, or to promote smoke-free environments. Previous reviews using BCTTv1 [32] to identify effective BCTs for smoking relapse in the postpartum period [36] and for smoking cessation in pregnancy [56] also found problem solving, information giving, and social support to be important. The most frequent BCT we identified in the present review was social support. Social support, particularly from partners, is recognised as a key barrier or facilitator in smoking cessation and remaining smoke free [16,57]. However, seven of our included trials [43,44,45,46,47,48,51] recruited only mothers or female caregivers. We found BCTs in the cluster of “goals and planning” to be most frequently used in our included effective interventions. This cluster includes advice on goal setting and strategies to overcome barriers to reach and maintain goals. Parents with younger infants, or with vulnerable children under paediatric care, or admitted to a NICU are under considerable acute and chronic stress [58,59,60,61], which likely acts as a barrier to creating and maintaining a smoke-free environment [15,16,20] and should be taken into consideration to aid goal setting and strategies to remain smoke free. Self-efficacy and ability to implement successful strategies is related to the BCT “verbal persuasion about capability” [32], which we identified as commonly occurring in effective interventions. For smoking parents, lower confidence to remain smoke-free, is a predictor of relapse [20], which this BCT may address. We identified information giving to be a key BCT to address smoking cessation, smoking relapse, or reduction of ETS. Parental smoking increases risk of child respiratory and other health conditions [2]. However, there are gaps in the knowledge base of parents and health professionals of the dangers of second-hand smoke [15,28,58] and how health professionals can effectively communicate these dangers to parents [15,28]. We found information provided from a “credible source” to be one of our “promising” BCTs. Belief of source credibility impacts attitudes and behaviour change, over and above attitudes about the validity of the information itself [62] and credibility may be particularly important for new parents, postpartum parents, or on admission of a child to paediatric care when parents are reliant on advice from health professionals.

Strengths of this review were undertaking comprehensive searches, full independent duplication of screening and data extraction, and the inclusion of a third reviewer to resolve any discrepancies. We included unpublished data from trials when made available by study authors.

Potential limitations to this review were incomplete reporting of BCTs in included studies. Study protocols or description of intervention designs were only available for seven trials [41,42,44,46,49,50,51], and these trials contained more BCTs. A review of BCTs in smoking cessation interventions has also found that fewer BCTs are described in published sources compared to those in unpublished data [63]. This may be particularly true for interventions using detailed components such as text message support [52]. We therefore took an inclusive approach to identifying BCTs, including those both probably (+) and definitely (++) present [32] to ensure any relevant BCTs were identified. We did not compare differences in BCTs across smoking behaviours (smoking cessation, smoking relapse prevention, or reduction in ETS) since studies largely targeted these behaviours together. BCTs within control conditions are particularly poorly described in published literature [63], and we did not compare BCTs in intervention groups with BCTs delivered to control groups, since so few BCTs were identified as being delivered exclusively to control groups. We did not conduct any statistical or subgroup analysis or assess which BCTs were associated with greater effect sizes, due to the small number of studies identified and diversity in populations, interventions, and outcomes reported [34,64]. Data synthesis was narrative and focused on components of effective interventions, an approach used in similar reviews [34,35,36,56]. We did not aim to compare BCTs within effective and non-effective trials; we aimed to explore which BCTs were common in effective interventions and which mechanisms of intervention delivery were commonly used, to give an indication of how BCTs might be best delivered, as a starting point to develop an intervention with optimal impact. There is no standard approach to identifying effective BCTs [34]. We defined “promising” BCTs as occurring in at least 25% of effective intervention studies [36]. We cannot definitively show any causal relationship with trial outcome for particular BCTs or mechanisms of delivery. However, the repeated presence of these components across effective interventions suggests these components might be the more promising ones to include in future interventions. In other words, “to identify the right intervention, for the right population at the right time”.

The majority of our trials were at low risk of bias, although we identified some feasibility concerns that might have limited our findings. It is likely there were additional feasibility issues that we were unaware of as reporting was inadequate in many trials. Most included trials were in high-income countries, but a third recruited from low-income areas, where smoking prevalence and exposure to ETS are likely to be higher [65]. We identified no U.K. trials. Most included trials were conducted in the U.S., where the healthcare system differs markedly from that in European countries. Previous reviews have found few smoking interventions in very vulnerable infants, such as NICU populations [27,30]. Indeed, only one of our included studies recruited specifically from a NICU [5]. We also found limited reporting of process measures within trials. The majority of trials included biochemical validation, but four [45,46,51,52] did not. We identified only one intervention using digital support in the form of text messages [52]. No other trials used newer harm reduction approaches such as e-cigarettes or other types of digital support (such as mobile apps), which have the potential to provide support in a more cost-effective manner. However, we identified one ongoing trial [53] that is using counselling in combination with nicotine replacement therapy, a mobile app, and texts; although this study is relatively small, aiming to recruit 149 participants per group. Many interventions to reduce ETS in children are short in duration and were therefore not included in this review. Further interventions incorporating newer approaches and holistic family support and with a duration of at least 6 months may be of benefit in the future. We recommend that studies better describe details of intervention mechanisms to enable further investigation of effective components, such as which BCTs would be most suited to particular populations.

## 5. Conclusions

There is a gap in knowledge regarding how best to reduce ETS exposure in young children, particularly for children in vulnerable groups. This review found that interventions effective in reducing ETS were delivered using counselling in combination with self-help materials and most commonly used BCTs involving education, goal setting and planning, and support to reach goals. Future interventions should consider these approaches to improve the chances of reducing child exposure to ETS, generating health and economic benefits for families and for wider society.

## Figures and Tables

**Table 1:** Frequency of behaviour change techniques (BCTs) identified in interventions to reduce environmental tobacco smoke.

BCT Code	BCT Label	BCT in Effective Interventions *n* (% Studies); Max *n* = 16
1.1	Goal setting (behaviour)	8 (50) *
1.2	Problem solving	11 (69) *
1.4	Action planning	8 (50) *
1.5	Review behaviour goal(s)	6 (38) *
1.6	Discrepancy between current behaviour and goal	1 (6)
1.7	Review outcome goal(s)	1 (6)
1.8	Behavioural contract	2 (13)
2.2	Feedback on behaviour	3 (19)
2.3	Self-monitoring of behaviour	3 (19)
2.6	Biofeedback	3 (19)
2.7	Feedback on outcome(s) of behaviour	1 (6)
3.1	Social support (unspecified)	13 (81) *
3.2	Social support (practical)	2 (13)
4.1	Instruction on how to perform a behaviour	7 (44) *
5.1	Information about health consequences	10 (63) *
5.2	Salience of consequences	1 (6)
5.3	Information about social and environmental consequences	4 (25) *
5.6	Information about emotional consequences	1 (6)
6.1	Demonstration of the behaviour	1 (6)
6.2	Social comparison	1 (6)
7.1	Prompts/cues	2 (13)
8.2	Behaviour substitution	4 (25) *
8.7	Graded tasks	1 (6)
9.1	Credible source	9 (56) *
9.2	Pros and cons	3 (19)
10.4	Social reward	7 (44) *
10.9	Self-reward	2 (13)
11.1	Pharmacological support	3 (19)
11.2	Reduce negative emotions	3 (19)
12.1	Restructuring the physical environment	2 (13)
12.2	Restructuring the social environment	2 (13)
12.3	Avoidance/reducing exposure to cues for the behaviour	2 (13)
12.5	Adding objects to the environment	5 (31) *
13.1	Identification of self as role model	1 (6)
13.2	Framing/reframing	2 (13)
13.3	Incompatible beliefs	1 (6)
13.5	Identity associated with changed behaviour	1 (6)
14.4	Reward approximation	3 (19)
15.1	Verbal persuasion about capability	4 (25) *
15.2	Mental rehearsal of successful performance	1 (6)
15.3	Focus on past success	2 (13)
16.2	Imaginary reward	1 (6)

* Effective BCT (in ≥25% studies).

## Supplementary Materials

The following are available online at https://www.mdpi.com/1660-4601/17/21/7731/s1, Table S1: References for articles of included trials, Table S2: Trial characteristics.

## References

[1] DoH (2017). Towards a Smokefree Generation. A Tobacco Control Plan for England.

[2] RCP (2010). Passive Smoking and Children.

[3] Chao M.R., Cooke M.S., Kuo C.Y., Pan C.H., Liu H.H., Yang H.J., Chen S.C., Chiang Y.C., Hu C.W. (2018). Children are particularly vulnerable to environmental tobacco smoke exposure: Evidence from biomarkers of tobacco-specific nitrosamines, and oxidative stress. Environ. Int..

[4] Hwang S.-H., Hwang J.H., Moon J.S., Lee D.-H. (2012). Environmental tobacco smoke and children’s health. Korean J. Pediatr..

[5] Blaakman S.W., Borrelli B., Wiesenthal E.N., Fagnano M., Tremblay P.J., Stevens T.P., Halterman J.S. (2015). Secondhand smoke exposure reduction after NICU discharge: Results of a randomized trial. Acad. Pediatr..

[6] Raghuveer G., White D.A., Hayman L.L., Woo J.G., Villafane J., Celermajer D., Ward K.D., de Ferranti S.D., Zachariah J. (2016). Cardiovascular consequences of childhood secondhand tobacco smoke exposure: Prevailing evidence, burden, and racial and socioeconomic disparities: A scientific statement from the American Heart Association. Circulation.

[7] Diver W.R., Jacobs E.J., Gapstur S.M. (2018). Secondhand smoke exposure in childhood and adulthood in relation to adult mortality among never smokers. Am. J. Prev. Med..

[8] Merianos A.L., Dixon C.A., Mahabee-Gittens E.M. (2017). Secondhand smoke exposure, illness severity, and resource utilization in pediatric emergency department patients with respiratory illnesses. J. Asthma.

[9] Schmitz J.E., Nwabuobi C.K., Pargas A., Camisasca-Lopina H., Sinkey R.G., Odibo A.O. (2019). Risk factors for neonatal intensive care unit admission among growth restricted fetuses [25P]. Obstet. Gynecol..

[10] Mason J., Wheeler W., Brown M.J. (2015). The economic burden of exposure to secondhand smoke for child and adult never smokers residing in U.S. public housing. Public Health Rep..

[11] Lam T.H., Leung G.M., Ho L.M. (2001). The effects of environmental tobacco smoke on health services utilization in the first eighteen months of life. Pediatrics.

[12] Vaz L.R., Jones M.J., Szatkowski L., Tata L.J., Petrou S., Coleman T. (2018). Estimating the health-care costs of children born to pregnant smokers in England: Cohort study using primary and secondary health-care data. Addiction.

[13] Adams E.K., Miller V.P., Ernst C., Nishimura B.K., Melvin C., Merritt R. (2002). Neonatal health care costs related to smoking during pregnancy. Health Econ..

[14] Godfrey C., Pickett K., Parrott S., Mdege N., Eapen D. (2010). Estimating the Costs to the NHS of Smoking in Pregnancy for Pregnant Women and Infants.

[15] Passey M.E., Longman J.M., Robinson J., Wiggers J., Jones L.L. (2016). Smoke-free homes: What are the barriers, motivators and enablers? A qualitative systematic review and thematic synthesis. BMJ Open.

[16] Notley C., Blyth A., Craig J., Edwards A., Holland R. (2015). Postpartum smoking relapse-a thematic synthesis of qualitative studies. Addiction.

[17] Livingstone-Banks J., Norris E., Hartmann-Boyce J., West R., Jarvis M., Chubb E., Hajek P. (2019). Relapse prevention interventions for smoking cessation. Cochrane Database Syst. Rev..

[18] Jones M., Lewis S., Parrott S., Wormall S., Coleman T. (2016). Re-starting smoking in the postpartum period after receiving a smoking cessation intervention: A systematic review. Addiction.

[19] Chamberlain C., O’Mara-Eves A., Porter J., Coleman T., Perlen S.M., Thomas J., McKenzie J.E. (2017). Psychosocial interventions for supporting women to stop smoking in pregnancy. Cochrane Database Syst. Rev..

[20] Orton S., Coleman T., Coleman-Haynes T., Ussher M. (2018). Predictors of postpartum return to smoking: A systematic review. Nicotine Tob. Res..

[21] Kanis J., Byczkowski T., Mahabee-Gittens E.M. (2014). Motivation to quit smoking in parental smokers in the pediatric emergency department. Pediatr. Emerg. Care.

[22] Bock B.C., Becker B.M., Borrelli B. (2008). Smoking behavior and risk perception among the parents of infants in the neonatal intensive care unit. Nicotine Tob. Res..

[23] McBride C.M., Lipkus I.M., Emmons K.M. (2003). Understanding the potential of teachable moments: The case of smoking cessation. Health Educ. Res..

[24] NICE (2013). Smoking: Acute, Maternity and Mental Health Services [PH48].

[25] NICE (2010). How to Stop Smoking in Pregnancy and Following Childbirth: Public Health Guideline [PH26].

[26] RCP (2018). Hiding in Plain Sight: Treating Tobacco Dependency in the NHS.

[27] Behbod B., Sharma M., Baxi R., Roseby R., Webster P. (2018). Family and carer smoking control programmes for reducing children’s exposure to environmental tobacco smoke. Cochrane Database Syst. Rev..

[28] Flemming K., Graham H., McCaughan D., Angus K., Sinclair L., Bauld L. (2016). Health professionals’ perceptions of the barriers and facilitators to providing smoking cessation advice to women in pregnancy and during the post-partum period: A systematic review of qualitative research. BMC Public Health.

[29] Nichols A., Clarke P., Notley C. (2019). Parental smoking and support in the NICU. Arch. Dis. Child. Fetal Neonatal Ed..

[30] Rosen L.J., Myers V., Winickoff J.P., Kott J. (2015). Effectiveness of interventions to reduce tobacco smoke pollution in homes: A systematic review and meta-analysis. IJERPH.

[31] Craig P., Dieppe P., Macintyre S., Michie S., Nazareth I., Petticrew M. (2008). Developing and evaluating complex interventions: The new Medical Research Council guidance. BMJ.

[32] Michie S., Richardson M., Johnston M., Abraham C., Francis J., Hardeman W., Eccles M.P., Cane J., Wood C.E. (2013). The behavior change technique taxonomy (v1) of 93 hierarchically clustered techniques: Building an international consensus for the reporting of behavior change interventions. Ann. Behav. Med..

[33] Moher D., Liberati A., Tetzlaff J., Altman D.G. (2009). Preferred reporting items for systematic reviews and meta-analyses: The PRISMA statement. BMJ.

[34] Michie S., West R., Sheals K., Godinho C.A. (2018). Evaluating the effectiveness of behavior change techniques in health-related behavior: A scoping review of methods used. Transl. Behav. Med..

[35] Lorencatto F., West R., Michie S. (2012). Specifying evidence-based behavior change techniques to aid smoking cessation in pregnancy. Nicotine Tob. Res..

[36] Brown T.J., Hardeman W., Bauld L., Holland R., Maskrey V., Naughton F., Orton S., Ussher M., Notley C. (2018). A systematic review of behaviour change techniques within interventions to prevent return to smoking postpartum. Addict. Behav..

[37] Higgins J.P.T., Thomas J., Chandler J., Cumpston M., Li T., Page M.J., Welch V.A. Cochrane Handbook for Systematic Reviews of Interventions Version 6.0 (updated July 2019). www.training.cochrane.org/handbook.

[38] Abdullah A.S., Hua F., Khan H., Xia X., Bing Q., Tarang K., Winickoff J.P. (2015). Secondhand smoke exposure reduction intervention in Chinese households of young children: A randomized controlled trial. Acad. Pediatr..

[39] Abdullah A.S., Mak Y.W., Loke A.Y., Lam T.H. (2005). Smoking cessation intervention in parents of young children: A randomised controlled trial. Addiction.

[40] Caldwell A.L., Tingen M.S., Nguyen J.T., Andrews J.O., Heath J., Waller J.L., Treiber F.A. (2018). Parental smoking cessation: Impacting children’s tobacco smoke exposure in the home. Pediatrics.

[41] Chan S.S.C., Cheung Y.T.D., Fong D.Y.T., Emmons K., Leung A.Y.M., Leung D.Y.P., Lam T.H. (2017). Family-based smoking cessation intervention for smoking fathers and nonsmoking mothers with a child: A randomized controlled trial. J. Pediatr..

[42] Emmons K.M., Hammond S.K., Fava J.L., Velicer W.F., Evans J.L., Monroe A.D. (2001). A randomized trial to reduce passive smoke exposure in low-income households with young children. Pediatrics.

[43] French G.M., Groner J.A., Wewers M.E., Ahijevych K. (2007). Staying smoke free: An intervention to prevent postpartum relapse. Nicotine Tob. Res..

[44] Greenberg R.A., Strecher V.J., Bauman K.E., Boat B.W., Fowler M.G., Keyes L.L., Denny F.W., Chapman R.S., Stedman H.C., LaVange L.M. (1994). Evaluation of a home-based intervention program to reduce infant passive smoking and lower respiratory illness. J. Behav. Med..

[45] Groner J.A., Ahijevych K., Grossman L.K., Rich L.N. (2000). The impact of a brief intervention on maternal smoking behavior. Pediatrics.

[46] Hannover W., Thyrian J.R., Roske K., Grempler J., Rumpf H.J., John U., Hapke U. (2009). Smoking cessation and relapse prevention for postpartum women: Results from a randomized controlled trial at 6, 12, 18 and 24 months. Addict. Behav..

[47] Hovell M.F., Zakarian J.M., Matt G.E., Hofstetter C.R., Bernert J.T., Pirkle J. (2000). Effect of counselling mothers on their children’s exposure to environmental tobacco smoke: Randomised controlled trial. BMJ.

[48] Hovell M.F., Zakarian J.M., Matt G.E., Liles S., Jones J.A., Hofstetter C.R., Larson S.N., Benowitz N.L. (2009). Counseling to reduce children’s secondhand smoke exposure and help parents quit smoking: A controlled trial. Nicotine Tob. Res..

[49] Lepore S.J., Collins B.N., Coffman D.L., Winickoff J.P., Nair U.S., Moughan B., Bryant-Stephens T., Taylor D., Fleece D., Godfrey M. (2018). Kids Safe and Smokefree (KiSS) multilevel intervention to reduce child tobacco smoke exposure: Long-term results of a randomized controlled trial. IJERPH.

[50] Ortega C.G., Peña C.C., Ortega J.A., Zafra M.S., Moreno J.L.B., Esteban J.A.P., Cuesta C.C., Martín-Cantera C., Cerezuela E.S., Pou R.M.C. (2015). Effectiveness of a brief primary care intervention to reduce passive smoking in babies: A cluster randomised clinical trial. J. Epidemiol. Community Health.

[51] Severson H.H., Andrews J.A., Lichtenstein E., Wall M., Akers L. (1997). Reducing maternal smoking and relapse: Long-term evaluation of a pediatric intervention. Prev. Med..

[52] Yu S., Duan Z., Redmon P.B., Eriksen M.P., Koplan J.P., Huang C. (2017). mHealth intervention is effective in creating smoke-free homes for newborns: A randomized controlled trial study in China. Sci. Rep..

[53] Collins B.N., Lepore S.J. (2017). Babies Living Safe & Smokefree: Randomized controlled trial of a multilevel multimodal behavioral intervention to reduce low-income children’s tobacco smoke exposure. BMC Public Health.

[54] Brown N., Luckett T., Davidson P.M., Di Giacomo M. (2015). Interventions to reduce harm from smoking with families in infancy and early childhood: A systematic review. IJERPH.

[55] Daly J.B., Mackenzie L.J., Freund M., Wolfenden L., Roseby R., Wiggers J.H. (2016). Interventions by health care professionals who provide routine child health care to reduce tobacco smoke exposure in children: A review and meta-analysis. JAMA Pediatrics.

[56] Campbell K.A., Fergie L., Coleman-Haynes T., Cooper S., Lorencatto F., Ussher M., Dyas J., Coleman T. (2018). Improving behavioral support for smoking cessation in pregnancy: What are the barriers to stopping and which behavior change techniques can influence these? Application of Theoretical Domains Framework. IJERPH.

[57] Flemming K., McCaughan D., Angus K., Graham H. (2015). Qualitative systematic review: Barriers and facilitators to smoking cessation experienced by women in pregnancy and following childbirth. J. Adv. Nurs..

[58] Adams K., Beem A., Diener E., Merritt T. (2012). Protecting the vulnerable: The importance of effective parental tobacco-dependence treatment during prenatal and newborn care. Pediatr. Allergy Immunol. Pulmonol..

[59] Ahlund S., Clarke P., Hill J., Thalange N.K. (2009). Post-traumatic stress symptoms in mothers of very low birth weight infants 2–3 years post-partum. Arch. Womens Ment. Health.

[60] Tsironi S., Koulierakis G. (2018). Factors associated with parents’ levels of stress in pediatric wards. J. Child. Health Care.

[61] Cousino M.K., Hazen R.A. (2013). Parenting stress among caregivers of children with chronic illness: A systematic review. J. Pediatr. Psychol..

[62] Schmidt A.M., Ranney L.M., Pepper J.K., Goldstein A.O. (2016). Source credibility in tobacco control messaging. Tob. Regul. Sci..

[63] de Bruin M., Black N., Javornik N., Viechtbauer W., Eisma M.C., Hartman-Boyce J., Williams A.J., West R., Michie S., Johnston M. (2020). Underreporting of the active content of behavioural interventions: A systematic review and meta-analysis of randomised trials of smoking cessation interventions. Health Psychol. Rev..

[64] Peters G.J., de Bruin M., Crutzen R. (2015). Everything should be as simple as possible, but no simpler: Towards a protocol for accumulating evidence regarding the active content of health behaviour change interventions. Health Psychol. Rev..

[65] ONS Adult Smoking Habits in the UK. https://www.ons.gov.uk/peoplepopulationandcommunity/healthandsocialcare/healthandlifeexpectancies/bulletins/adultsmokinghabitsingreatbritain/2018#characteristics-of-current-cigarette-smokers-in-the-uk.

