# Supplementary material for: Systematic Review of Behaviour Change Techniques within Interventions to Reduce Environmental Tobacco Smoke Exposure for Children
Source: Int J Environ Res Public Health. 2020 Oct 22;17(21):7731. doi: 10.3390/ijerph17217731 (PMC7660048; doi:10.3390/ijerph17217731)
Supplement: Supplementary file 1 [file ijerph-17-07731-s001.zip › Table S1.docx]

**Supplementary Table 1: References for articles of included trials**

| **Main trial** | **Trial references** |
| --- | --- |
| Abdullah 2015 | Abdullah AS, Hua F, Khan H, et al. Secondhand smoke exposure reduction intervention in Chinese households of young children: a randomized controlled trial. *Acad Pediatr.* 2015;15:588-598. |
| Abdullah 2005 | *Abdullah AS, Mak YW, Loke AY, Lam TH. Smoking cessation intervention in parents of young children: a randomised controlled trial. *Addiction.* 2005;100:1731-1740.  Abdullah ASM, Lam TH, Mak YW, Loke AY. A randomized control trial of a smoking cessation intervention on parents of young children- a preliminary report. Society for Research on Nicotine and Tobacco 10th Annual Meeting; 18-21 February, 2005; Phoenix, AZ. |
| Blaakman 2015 | Blaakman SW, Borrelli B, Wiesenthal EN, et al. Secondhand smoke exposure reduction after NICU discharge: results of a randomized trial. *Acad Pediatr.* 2015;15:605-612. |
| Caldwell 2018 | *Caldwell AL, Tingen MS, Nguyen JT, et al. Parental smoking cessation: impacting children's tobacco smoke exposure in the home. *Pediatrics.* 2018;141:S96-s106.  Tingen MS, Andrews JO, Heath J, Turnmire AE, Waller JL, Treiber FA. Comparison of enrollment rates of African-American families into a school-based tobacco prevention trial using two recruitment strategies in urban and rural settings. *Am J Health Promot.* 2013;27:e91-e100. |
| Chan 2017 | Chan S. Family intervention to reduce second hand smoke (SHS) exposure in children. ISRCTN registry 2011. Available at: <https://doi.org/10.1186/ISRCTN99111655> (accessed 18th November 2019).  Chan SSC, Lam TH, Leung GM, et al. *A Proactive Family Smoking Cessation Intervention for Parents of Children 0-18 Months: A Randomized Controlled Trial*. RFCID/HHSRF/HSRF/HCPF; 2011; Hong Kong.  *Chan SSC, Cheung YTD, Fong DYT, et al. Family-based smoking cessation intervention for smoking fathers and nonsmoking mothers with a child: a randomized controlled trial. *J Pediatr.* 2017;182: 260-266.  Cheung YTD, Wang MP, Chan SSC, Lam TH. Helping smoking fathers with an infant to quit: the family-based intervention impact on marital satisfaction and partner support to quit. The 22nd Annual Meeting of the Society for Research on Nicotine and Tobacco; 2-5 March, 2016; Chicago, IL. |
| Emmons 2001 | *Emmons KM, Hammond SK, Fava JL, Velicer WF, Evans JL, Monroe AD. A randomized trial to reduce passive smoke exposure in low-income households with young children. *Pediatrics.* 2001;108: 18-24.  Emmons KM, Wong M, Hammond SK, et al*.* Intervention and policy issues related to children's exposure to environmental tobacco smoke. *Prev Med.* 2001;32:321-331. |
| French 2007 | French GM, Groner JA, Wewers ME, Ahijevych K. Staying smoke free: an intervention to prevent postpartum relapse. *Nicotine Tob Res.* 2007;9:663-670. |
| Greenberg 1994 | *Greenberg RA, Strecher VJ, Bauman KE, et al. Evaluation of a home-based intervention program to reduce infant passive smoking and lower respiratory illness. *J Behav Med.* 1994;17:273-290.  Margolis PA, Keyes LL, Greenberg RA, Bauman KE, LaVange LM. Urinary cotinine and parent history (questionnaire) as indicators of passive smoking and predictors of lower respiratory illness in infants. *Pediatr Pulmonol.* 1997; 23:417-423.  Strecher VJ, Bauman KE, Boat B, Fowler MG, Greenberg RA, Stedman H. The development and formative evaluation of a homebased intervention to reduce passive smoking by infants. *Health Educ Res.* 1989;4:225-232.  Strecher VJ, Bauman KE, Boat B, Fowler MG, Greenberg R, Stedman H. The role of outcome and efficacy expectations in an intervention designed to reduce infants' exposure to environmental tobacco smoke. *Health Educ Res.* 1993;8:137-143. |
| Groner 2000 | Groner JA, Ahijevych K, Grossman LK, Rich LN. The impact of a brief intervention on maternal smoking behavior. *Pediatrics.* 2000;105:267-271. |
| Hannover 2009 | *Hannover W, Thyrian JR, Roske K, et al*.* Smoking cessation and relapse prevention for postpartum women: results from a randomized controlled trial at 6, 12, 18 and 24 months. *Addict Behav.* 2009;34:1-8.  Roske K, Hannover W, Grempler J, et al*.* Post-partum intention to resume smoking. *Health Educ Res.* 2006;21:386-392.  Roske K, Schumann A, Hannover W, et al*.* Postpartum smoking cessation and relapse prevention intervention: a structural equation modeling application to behavioral and non-behavioral outcomes of a randomized controlled trial. *J Health Psychol.* 2008;13:556-568.  Röske K, Hannöver W, Thyrian JR, John U, Hannich H-J. Smoking cessation counselling for pregnant and postpartum women among midwives, gynaecologists and paediatricians in Germany. *IJERPH.* 2009;6:96-107.  Thyrian JR, Hannover W, Grempler J, Roske K, John U, Hapke U. An intervention to support postpartum women to quit smoking or remain smoke-free. *J Midwifery Womens Health.* 2006;51:45-50.  Thyrian JR, Hannover W, Roske K, Rumpf HJ, John U, Hapke U. Postpartum return to smoking: identifying different groups to tailor interventions. *Addict Behav.* 2006;31:1785-1796.  Thyrian JR, Freyer-Adam J, Hannover W, et al*.* Adherence to the principles of motivational interviewing, clients' characteristics and behavior outcome in a smoking cessation and relapse prevention trial in women postpartum. *Addict Behav.* 2007;32:2297-2303.  Thyrian JR, Freyer-Adam J, Hannover W, et al. Population-based smoking cessation in women post partum: adherence to motivational interviewing in relation to client characteristics and behavioural outcomes. *Midwifery.* 2010;26:202-210. |
| Hovell 2000 | *Hovell MF, Zakarian JM, Matt GE, Hofstetter CR, Bernert JT, Pirkle J. Effect of counselling mothers on their children's exposure to environmental tobacco smoke: randomised controlled trial. *BMJ.* 2000;321:337-342.  Hovell MF, Zakarian JM, Matt GE, Hofstetter CR, Bernert JT, Pirkle J. Decreasing environmental tobacco smoke exposure among low income children: preliminary findings. *Tob Control.* 2000;9 Suppl 3:Iii70-71. |
| Hovell 2009 | *Hovell MF, Zakarian JM, Matt GE, et al*.* Counseling to reduce children's secondhand smoke exposure and help parents quit smoking: a controlled trial. *Nicotine Tob Res.* 2009;11:1383-1394.  Liles S, Hovell MF, Matt GE, Zakarian JM, Jones JA. Parent quit attempts after counseling to reduce children's secondhand smoke exposure and promote cessation: main and moderating relationships. *Nicotine Tob Res.* 2009;11:1395-1406. |
| Lepore 2018 | Collins BN, Nair US, Godfrey M. Kids Safe and Smokefree: a multilevel trial to protect children from tobacco smoke and promote cessation among low-income parents. Society for Research in Nicotine and Tobacco Europe; 10-12 September, 2015; Maastricht, Netherlands.  Collins BN, Lepore SJ, Winickoff JP, et al. An office-initiated multilevel intervention for tobacco smoke exposure: a randomized trial. *Pediatrics.* 2018;141:S75-S86.  Lepore SJ, Winickoff JP, Moughan B, et al. Kids Safe and Smokefree (KiSS): a randomized controlled trial of a multilevel intervention to reduce secondhand tobacco smoke exposure in children. *BMC Public Health.* 2013;13:792.  *Lepore SJ, Collins BN, Coffman DL, et al. Kids Safe and Smokefree (KiSS) multilevel intervention to reduce child tobacco smoke exposure: long-term results of a randomized controlled trial. *IJERPH.* 2018;15: doi: 10.3390/ijerph15061239. |
| Ortega 2015 | Ortega G, Castellà C, Martín-Cantera C, et al. Passive smoking in babies: The BIBE study (brief intervention in babies. effectiveness). *BMC Public Health.* 2010;10:772.  *Ortega CG, Peña CC, Ortega JA, et al. Effectiveness of a brief primary care intervention to reduce passive smoking in babies: a cluster randomised clinical trial. *J Epidemiol Community Health.* 2015;69:249. |
| Severson 1997 | Severson HH, Zoref L, Andrews J, Lichtenstein E, Wall M. Reducing environmental tobacco smoke (ETS) exposure for infants: a cessation intervention for mothers of newborns. *Am J Health Promot.* 1994;8:252-253.  Severson HH, Andrews JA, Lichtenstein E, Wall M, Zoref L. Predictors of smoking during and after pregnancy: a survey of mothers of newborns. *Prev Med.* 1995;24:23-28.  *Severson HH, Andrews JA, Lichtenstein E, Wall M, Akers L. Reducing maternal smoking and relapse: long-term evaluation of a pediatric intervention. *Prev Med.* 1997;26:120-130.  Wall MA, Severson HH, Andrews JA, Lichtenstein E, Zoref L. Pediatric office-based smoking intervention: impact on maternal smoking and relapse. *Pediatrics.* 1995;96:622-628. |
| Yu 2017 | Yu S, Duan Z, Redmon PB, Eriksen MP, Koplan JP, Huang C. mHealth intervention is effective in creating smoke-free homes for newborns: a randomized controlled trial study in China. *Sci Rep.* 2017;7:9276. |

* Main trial reference
